# Characterization of yeast histone H3-specific type B histone acetyltransferases identifies an *ADA2*-independent Gcn5p activity

**DOI:** 10.1186/1471-2091-5-11

**Published:** 2004-07-26

**Authors:** Amy R Sklenar, Mark R Parthun

**Affiliations:** 1 Department of Molecular and Cellular Biochemistry, College of Medicine and Public Health, The Ohio State University, Columbus, OH 43210, USA

## Abstract

**Background:**

The acetylation of the core histone NH_2_-terminal tails is catalyzed by histone acetyltransferases. Histone acetyltransferases can be classified into two distinct groups (type A and B) on the basis of cellular localization and substrate specificity. Type B histone acetyltransferases, originally defined as cytoplasmic enzymes that acetylate free histones, have been proposed to play a role in the assembly of chromatin through the acetylation of newly synthesized histones H3 and H4. To date, the only type B histone acetyltransferase activities identified are specific for histone H4.

**Results:**

To better understand the role of histone acetylation in the assembly of chromatin structure, we have identified additional type B histone acetyltransferase activities specific for histone H3. One such activity, termed HatB3.1, acetylated histone H3 with a strong preference for free histones relative to chromatin substrates. Deletion of the GCN5 and ADA3 genes resulted in the loss of HatB3.1 activity while deletion of ADA2 had no effect. In addition, Gcn5p and Ada3p co-fractionated with partially purified HatB3.1 activity while Ada2p did not.

**Conclusions:**

Yeast extracts contain several histone acetyltransferase activities that show a strong preference for free histone H3. One such activity, termed HatB3.1, appears to be a novel Gcn5p-containing complex which does not depend on the presence of Ada2p.

## Background

Histones H3 and H4 are among the most evolutionarily conserved proteins (>90% identity from yeast→humans) [1]. Octamers composed of one histone H3/H4 tetramer and two histone H2A/H2B dimers package 146 bp of DNA into the basic repeating subunit of chromatin, the nucleosome [1]. Hence, as fundamental components of chromatin, these proteins are an integral part of all cellular processes involving chromosomal DNA.

The physical characteristics of the histones are precisely regulated in the cell by an elaborate network of post-translational modifications that include acetylation, methylation, phosphorylation, ubiquitination and ADP-ribosylation [2-4]. These modifications are found primarily on the NH_2_-terminal tails of the histones. These domains, which protrude from the core of the nucleosome, are free to interact with, and be acted upon by, the nuclear environment. The past several years has seen the identification of numerous enzymes that are capable of modifying the histones. These enzymes are generally found in large, multi-subunit complexes and have activities that are not only specific for a given histone but are specific for particular amino acid residues within the histone [5,6].

The most well characterized histone modifying enzymes are the histone acetyltransferases (HATs). HATs catalyze the transfer of an acetyl moiety from acetyl-coenzyme A to the ε-amino group of lysine residues in the histone NH_2_-terminal tails. Historically, these enzymes have been classified as either type A or type B, based upon substrate specificity and cellular localization [7]. Found in the nucleus, type A HATs utilize nucleosomal histones as substrates. A number of Type A HATs have been identified in yeast. These include Gcn5p (SAGA, ADA, SLIK, SALSA and HAT-A2 complexes), Sas2p (SAS complex), Sas3p (NuA3 complex), Esa1p (NuA4 and picNuA4 complexes) and Elp3 (Elongator complex) [8-22]. These enzymes have been characterized primarily in the context of transcriptional activation but are likely to be involved in other chromatin mediated events as well [23,24].

Type B HATs were initially described as cytoplasmic enzymes that acetylate free histones in conjunction with chromatin assembly [7]. The *de novo *assembly of chromatin is a complex, multi-step process that occurs most prominently during DNA replication (but also accompanies other cellular processes involving DNA synthesis) [25,26]. Following induction of histone mRNA synthesis, histone proteins are translated in the cytoplasm. For histones H3 and H4, synthesis is rapidly followed by the acetylation of specific lysine residues in their NH_2_-terminal tail domains [27]. For newly synthesized histone H4, this acetylation occurs on lysine residues at positions 5 and 12 in all eukaryotic organisms examined to date [28,29]. For newly synthesized histone H3, acetylation appears to occur in distinct patterns that can differ from organism to organism [28,30,31]. The acetylated H3 and H4 form tetramers that are translocated into the nucleus and loaded onto DNA [32]. Following completion of the histone octamer by histone H2A/H2B addition, mature chromatin is formed following the deacetylation of histones H3 and H4 [33,34].

In contrast to the type A HATs, only one type B HAT has been characterized to date, Hat1p. Hat1p is an evolutionarily conserved enzyme that specifically acetylates free histone H4 [35-38]. Consistent with its identification as a type B HAT, recombinant yeast Hat1p, as well the Xenopus and Human Hat1p homologs, acetylates both lysine 5 and lysine 12 [35-39]. Hat1p was originally purified from yeast cytoplasmic extracts in a complex with Hat2p, a yeast homolog of the mammalian Rbap46/48 proteins [36,40,41]. Subsequent studies have shown that yeast Hat1p, as well as its higher eukaryotic counterparts, can also localize to the nucleus [37,38,42]. These results suggest that, while specificity for free histones is a bona fide characteristic, cytoplasmic localization may not be a strict criterion for classification as a type B HAT.

Evidence has accumulated indicating that the acetylation of newly synthesized histones H3 and H4 play over-lapping roles in chromatin assembly. While yeast strains carrying a deletion of either the H3 or H4 NH_2_-terminal tail are viable, concomitant deletion of both NH_2_-termini (or combining tail deletions with alterations in specific sites of acetylation) results in a defect in nucleosome assembly and cell death [43,44]. In addition, while deletion of the *HAT1 *gene produces no observable phenotype, combining a deletion of *HAT1 *with specific lys→arg mutations in the NH_2_-terminus of histone H3 generates defects in both telomeric silencing and DNA damage repair [45,46]. However, despite the importance of the acetylation of newly synthesized histone H3 in chromatin assembly, there have been no type B histone acetyltransferases described that specifically target histone H3.

To identify potential histone H3-specific type B HATs, we have systematically surveyed yeast extracts for candidate activities. Here we detail one such activity, termed HatB3.1. We provide evidence that this is a novel complex that utilizes Gcn5p as its catalytic subunit. Intriguingly, unlike previously identified Gcn5p-containing HAT complexes, HatB3.1 contains Ada3p, but not Ada2p.

## Results

### Identification of histone H3-specific type B histone acetyltransferase activities in yeast

The highly selective activity of the native Hat1p/Hat2p complex for free versus nucleosomal histone H4 is the primary characteristic that distinguishes this enzyme from the type A histone acetyltransferases [35,36]. Therefore, to identify putative histone H3-specific type B HAT complexes, we systematically surveyed yeast extracts for activities that acetylated free histone H3 but not histone H3 packaged into chromatin. Extracts were prepared from cell cultures grown to mid-log phase to enrich for actively dividing cells, as the most robust period of chromatin assembly occurs during DNA replication. Yeast cell walls were digested with zymolyase and cytosolic extracts were produced by the lysis of the cells in low salt buffer followed by centrifugation to remove nuclei and large cell debris. Hence, this extract contained soluble cytoplasmic proteins as well as proteins loosely associated with the nucleus. The nuclear extract was obtained by incubating the nuclear pellet in buffer containing 1.0 M NaCl to extract proteins that are more tightly associated with the nucleus.

It is difficult to reliably detect histone acetyltransferase activities in the relatively crude cytosolic and nuclear extracts. Therefore, to evaluate the intrinsic HAT activities present in each of the extracts, they were fractionated by anion and cation exchange chromatography. Fractions were assayed for HAT activity using ^3^H-acetyl Coenzyme A and equivalent amounts of either free histones or chromatin as substrate. Histones were then resolved by SDS-PAGE and acetylated species visualized by fluorography.

**Figure 1:**
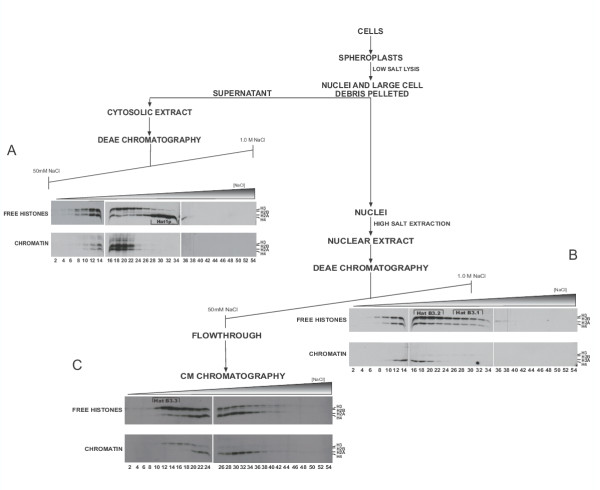
**Identification of putative histone H3-specific type B histone acetyltransferases. **Cytosolic and nuclear extracts were prepared and fractionated as outlined in the flow chart. Inherent HAT activities were identified by assaying column fractions with ^3^H-Acetyl-Coenzyme A and equivalent amounts of either free histones or chromatin (as indicated). Reaction products were resolved by 18% SDS-PAGE and visualized by fluorography. The relative migrations of the core histones, as determined from coomassie blue staining, are denoted at the side of each fluorogram. The positions of Hat1p and putative H3-specific type B HATs are indicated by brackets.

Fractionation of the cytosolic extract on a DEAE column is shown Figure 1A. As expected, the predominant type B activity present in these preparations was attributable to Hat1p, as indicated by robust, free histone H4 acetylation (Fig. 1A, lanes 28–34). The identity of the Hat1p/Hat2p complex was confirmed by western blot analysis using polyclonal antibodies against both Hat1p and Hat2p (data not shown).

The cytosolic extract also contained at least two additional HAT activities. The first showed a clear peak that was centered on fraction 14 and acetylated free histones H3, H2B and H4. The activity of this HAT on chromatin was more difficult to determine as the H3 and H4 labeling seen in these fractions does not show a marked peak in fraction 14 and may be due to the leading edge of a HAT activity eluting at higher salt. Therefore, this activity may be a candidate type B HAT. There was also a distinct peak of HAT activity at fractions 18–20. With free histone substrates, this activity primarily acetylated histone H3. However, there was also a coincident peak of chromatin H3 and H4 acetylating activity in these fractions suggesting this activity is likely to be a type A HAT.

**Figure 2:**
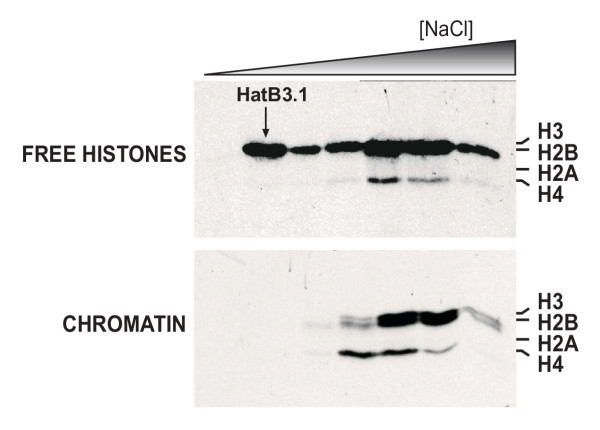
**HatB3.1 is a chromatographically distinct activity. **DEAE fractions encompassing HatB3.1 activity were pooled and subjected to further fractionation. Fluorograms of liquid HAT assays, using free histones or chromatin as substrates, representative of gradient eluted Mono Q column fractions, showed that HatB3.1 activity can be separated from the overlapping activities present in the initial DEAE fractionation. Migration of the core histones is indicated. Migration of free histone H3 activity attributable to HatB3.1 is marked with an arrow.

**Figure 3:**
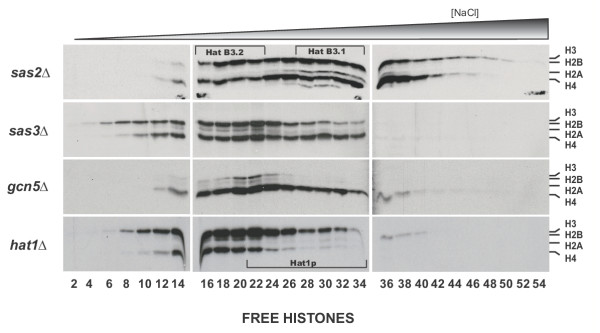
**HatB3.1 activity is dependent upon GCN5. **Nuclear extracts, generated as depicted in Figure 1 from the indicated isogenic deletion strains, were fractionated via DEAE anion exchange chromatography. Fluorograms of HAT assays resolved by 18% SDS-PAGE are shown with the migration of the core histones as indicated. Fractions of equivalent conductance are aligned for each strain. Regions containing HatB3.1, HatB3.2 and Hat1p are identified by brackets.

**Figure 4:**
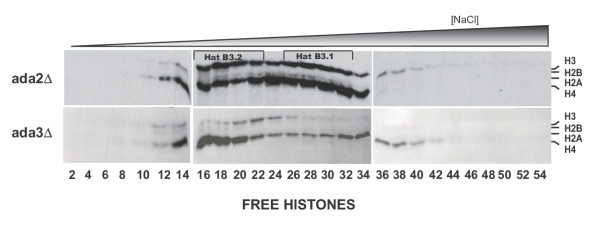
**Highly purified HatB3.1 contains Gcn5p and Ada3p in a high molecular weight complex. **A) Flowchart outlining the partial purification of HatB3.1. B) Fluorograms of liquid HAT assays of the Superose 6 column fractionation of HatB3.1 activity (top 2 panels). Assays used either free histones or chromatin as substrate (as indicated). The relative elution of molecular weight standards is shown along the top, while the migration of histones H3 and H4 is indicated at the right. Column fraction aliquots (15 μL) were also resolved by SDS-PAGE and visualized by silver staining (bottom panel, protein ladder mobility is represented at right). Corresponding fraction numbers for both the fluorograms and silver stained gel are indicated along the bottom. C) Peak HatB3.1 containing Superose 6 fractions, as indicated at top of blots, were resolved by three identical 10% SDS gels, transferred to nitrocellulose and probed with the indicated antibodies (left of blots). Presence of Gcn5p, Ada2p and Ada3p in nuclear extract and/or column fractions was visualized via chemifluoresence. Relative migration of protein standards is shown on the right.

DEAE fractionation of the nuclear extract also revealed several distinct HAT activities (Figure 1B). There were two H4-specific type A HAT activities that peaked at fractions 14 and 18, as indicated by activity on both free and nucleosomal histones. There was also a significant peak of activity that acetylated free histone H3 (there was also slight acetylation of histone H2B that is more easily seen in Figure 3) that was coincident with a minor nucleosomal H3 HAT activity (Fig. 1B right panel, lanes 16–34). This activity also partly overlapped the nucleosomal H4 activities. This peak of activity was rather broad and most probably results from the partial overlap of at least two distinct activities. In fact, the separation of these activities was readily apparent in Figures 3 and 4. The strong overall preference of these activities for free histone H3 makes them good candidates for H3-specific type B HATs. As these are chromatographically distinct activities we have termed them HatB3.1 and HatB3.2 as indicated (Figure 1B, right panel).

Unbound material from the initial DEAE fractionation of the cytosolic and nuclear extracts was analyzed by cation exchange chromatography (carboxymethyl sepharose (CM)). While this fraction from the cytosolic extract appeared inactive, there were several additional HAT activities resolved from the nuclear extract (Figure 1C, data not shown). The presence of these activities in the DEAE flowthrough fraction is not simply due to column overloading as recycling the flowthrough fraction over the DEAE column a second time did not result in significant protein retention. Hence, these activities are chromatographically distinct from those that bind the DEAE resin. Two activities, centered on fractions 22 and 30, acetylated primarily histone H4. These appeared to be typical type A HAT's as they were active on both free histones and chromatin. A broad peak of histone H3-specific activity eluted from the CM column from fraction 10 through fraction 24 (with activity trailing through the remainder of the gradient). Comparison of the free histone and chromatin activities in these fractions suggested that this region of the gradient actually contained overlapping type A and type B activities. There was a distinct peak of free histone H3 acetylating activity centered on fractions 12 – 14 while acetylation of chromatin associated H3 peaked in fraction 16. Hence, the activity in fractions 12–14 is another candidate H3-specific type B HAT (labeled HatB3.3).

### HatB3.1 is specific for free histone H3

The fractions from the DEAE column that contained the activity that we have termed HatB3.1 modified not only free histone H3 but also free H4. In addition, a low level of nucleosomal H3 activity could also be seen in these fractions. To determine whether these activities were the result of a single enzyme complex or were due to multiple, overlapping complexes, these fractions were pooled, dialyzed and fractionated over a Mono-Q column (Figure 2). Inspection of the HAT activity profile of the fractions eluting from the Mono-Q column clearly demonstrated that multiple HAT activities overlapped with HatB3.1 during the initial fractionation of the nuclear extract. The HatB3.1 activity eluted from the Mono-Q column very early in the gradient and appeared to be highly specific for free histone H3. The second activity to elute from the Mono-Q column was specific for chromatin-associated histone H4. The third activity acetylated both free and nucleosomal histones H3, H2B and H4. These results indicated that the acetylation of multiple histones in the DEAE elution profile was the result of at least three overlapping activities and confirmed that HatB3.1 is a chromatographically distinct free histone H3-specific activity. Therefore, HatB3.1 was a good candidate for further characterization.

### HatB3.1 activity is dependent on GCN5

To gain insight into the identity of the catalytic subunit of HatB3.1, we constructed null mutants for each of the yeast HAT's that have demonstrated histone H3 activity as well as the known type B HAT, *HAT1*. Isogenic deletion strains (Δ*gcn5*, Δ*sas2*, Δ*sas3 *and Δ*hat1*) were grown and protein extracts prepared exactly as for the wild type strain. Nuclear extracts were again fractionated via DEAE column chromatography and fractions of equivalent conductivity assayed for HAT activity as described above.

Parallel comparison of the HAT activity profiles from each strain provided biochemical evidence for the dependency of specific histone acetyltransferase activities on the presence of a particular HAT catalytic subunit (compare Figure 3 with Figure 1B). While subtle variations in observed specificity and intensity of HAT activity were seen throughout the profiles of the Δ*sas2 *and Δ*sas3 *strains, the robust H3 acetylation attributed to the HatB3.1 activity appeared unaffected by deletion of these enzymes (Figure 3, lanes 26–34). Conversely, HatB3.1 activity was abolished in a Δ*gcn5 *strain (Figure 3, lanes 26–34). In addition, the HatB3.2 activity also appeared to be absent in extracts from a gcn5 strain indicating that both of these putative type B HAT activities are dependent on Gcn5p. Additionally, the integrity, in a Δ*gcn5 *strain, of the overlapping free histone and chromatin (data not shown) activities in this region of the gradient confirmed that HatB3.1 was a chromatographically distinct HAT activity exhibiting specificity for free histone H3.

Analysis of the activity profile from nuclear extracts derived from a Δ*hat1 *strain identified a broad peak of Hat1p dependent activity that spanned fractions ~22–34. Western blot analysis using antibodies against Hat1p and Hat2p confirmed the presence of these proteins in fractions from this region of the gradient from the wild type extract (data not shown). As with the Hat1p-dependent activity in cytosolic extracts, this activity also appeared to be specific for free histone H4. This result confirmed previous observations indicating that Hat1p is localized to both the cytoplasm and the nucleus [37,42]. In addition, the presence of an authentic type B HAT activity in our nuclear extracts validated our use of these extracts for the identification of putative histone H3-specific type B HAT activities.

### HatB3.1 activity is dependent on ADA3 but not ADA2

There are two proteins, Ada2p and Ada3p, that are components of all known Gcn5p-containing HAT complexes and that are required for the activity of these complexes [9-11,13,14]. To determine whether the HatB3.1 activity was also dependent on these proteins, nuclear extracts were prepared from isogenic Δ*ada2 *and Δ*ada3 *strains and the status of the HatB3.1 activity determined by DEAE chromatography. As shown in Figure 4, the loss of *ADA2 *did not affect either the HatB3.1 or HatB3.2 activity but did cause a substantial increase in the free histone H4 specific activity that eluted late in the DEAE gradient. However, the HAT activity profile of the Δada3 extracts was strikingly similar to that seen for the gcn5 extracts with both the HatB3.1 and HatB3.2 activities absent. These results indicated that the HatB3.1 activity was dependent on *ADA3 *and that Ada2p is either not a component of the HatB3.1 activity or is not required for its stability.

### Partial purification of HatB3.1

**Figure 5:**
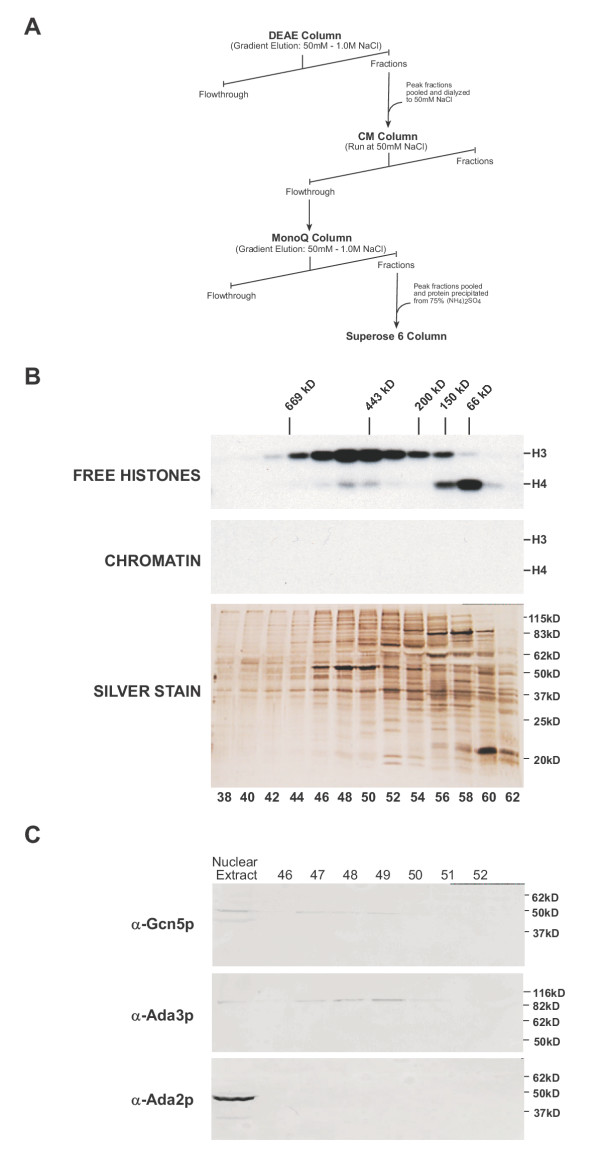
**ADA3, but not ADA2, is essential for HatB3.1 activity. **Nuclear extracts from isogenic Δada2 and Δada3 strains were prepared and fractionated as previously described for wild type and HAT deletion strains (Figures 1 and 3). Fluorograms reflecting HAT activity assays from fractions of equivalent conductivity from each strain, using free histones, are shown (fraction numbers are displayed at bottom [compare lanes to those in figure 3 as well]). Regions of HatB3.1 and HatB3.2 are highlighted by brackets.

To further characterize HatB3.1, this activity was purified through several chromatographic steps. The purification scheme is diagramed in Figure 5A. HatB3.1 containing fractions from the DEAE column were pooled, dialyzed to a conductivity similar to that of the loading buffer (DN(50)) and the dialysate applied to a cation exchange column (CM sepharose). HAT activity assays indicated that the HatB3.1 activity flowed through the CM sepharose column while bound proteins, resolved by a linear salt gradient, contained co-purifying HAT activities that acetylated both free and nucleosomal, H3 and H4 (data not shown). The presence of HatB3.1 in the CM sepharose flow through also confirmed that HatB3.1 and HatB3.3 were distinct activities.

The proteins that flowed through the CM sepharose column were applied to a Mono-Q column and then eluted with a linear salt gradient. Fractions containing free histone H3 activity were pooled and concentrated by precipitation with 75% ammonium sulfate. The sample was then fractionated by size exclusion chromatography using a Superose 6 column. As seen in Figure 5B, the HatB3.1 activity peaked at fractions 48–50, indicating that a high molecular weight complex of ~500 kDa was responsible for this activity. The size of HatB3.1 remained stable throughout the course of purification as Superose 6 fractionation of the pooled HatB3.1 activity from the initial DEAE column displayed an identical mass (data not shown). The highly purified HatB3.1 retained its high degree of specificity for free histone versus chromatin substrates. There were also two peaks of free histone H4 specific activity seen in the Superose 6 elution profile. Western blot analysis indicated that Hat1p co-eluted with the low molecular weight species. The second peak of H4 activity co-purified with HatB3.1. Whether this acetylation of histone H4 was the result of a weak specificity of HatB3.1 for H4 or due to a second, co-eluting, HAT activity has not been resolved.

### Gcn5p and Ada3p, but not Ada2p, co-purified with the HatB3.1 activity

The absence of HatB3.1 activity in extracts from a Δgcn5 strain indicated that HatB3.1 was dependent on Gcn5p, either indirectly via Gcn5p-mediated transcriptional regulation, or directly, as its catalytic subunit. While the HatB3.1 activity was highly purified relative to the initial nuclear extract, the peak Superose 6 fractions were still too complex to allow the definitive identification of specific bands that co-purified with the activity (Figure 5B). Extensive efforts to purify HatB3.1 to homogeneity have been unsuccessful. To determine whether Gcn5p was likely to be functioning as the catalytic subunit of HatB3.1, fractions across the peak of HatB3.1 activity from the Superose 6 column were probed with anti-Gcn5p antibodies. As seen in Figure 5C, Gcn5p was present in the fractions containing the peak of HatB3.1 activity from the Superose 6 column. This result is consistent with direct association of Gcn5p with the HatB3.1 complex.

Duplicate blots were probed with anti-Ada2p and anti-Ada3p antibodies to determine whether these proteins also co-fractionated with the HatB3.1 complex. As expected, both Ada2p and Ada3p are present in the nuclear extracts (Figure 5C). However, while Ada3p precisely co-purified with Gcn5p and the peak of HatB3.1 activity, Ada2p did not appear to be associated with this complex. The absence of the Ada2p from the peak of HatB3.1 activity is consistent with the observation that HatB3.1 activity is independent of the ADA2 gene and suggests that Ada2p is not a component of the HatB3.1 complex. The absence of an Ada2p signal on the Western blot was not due to problems with sensitivity as comparison of the relative signals of Gcn5p, Ada2p and Ada3p in the nuclear extracts and Superose 6 fractions demonstrated that the presence of Ada2p in the Superose 6 fractions would have been readily apparent. While the HatB3.1 activity is enriched in the Superose 6 peak fractions relative to the original nuclear extract, the amount of Gcn5p and Ada3p present in these fractions is not enriched relative to the nuclear extract due to the fact that these proteins are components of at least five other histone acetyltransferase complexes. Hence, only a fraction of the Gcn5p and Ada3p present in the cell extracts was associated with HatB3.1.

## Discussion

Considerable genetic and biochemical evidence indicates that, in most organisms, newly synthesized histone H3 is acetylated and that this acetylation plays a role in the *de novo *assembly of chromatin [28,30,31,43-48]. However, the enzymes responsible for this modification have remained elusive. In the present study we have comprehensively surveyed yeast extracts for putative, histone H3-specific, type B histone acetyltransferase activities. At least three candidate activities were identified, HatB3.1, HatB3.2 and HatB3.3. Further characterization of HatB3.1 indicated that this activity is a novel ~500 kDa HAT complex. In addition, our results suggest that Gcn5p and Ada3p are components of this complex but that, contrary to all previously isolated Gcn5p complexes, HatB3.1 is not associated with Ada2p. It does not appear that the HatB3.1 complex is merely an unstable form of one of the previously characterized Gcn5p-containing complexes as the apparent molecular weight of HatB3.1 did not vary during the course of its purification.

There have been at least a dozen distinct HAT complexes identified in yeast [8-22,36,42]. Conservative analysis of our systematic fractionation of yeast cytosolic and nuclear extracts resolved 12 chromatographically separable activities. However, many of these activities were represented by rather broad peaks, likely to be composed of partially overlapping activities that may differentiate upon further purification (as seen in Figure 2). While many of the activities identified here may correspond to previously characterized complexes, it is difficult to determine these relationships, as our initial purification steps differ from those typically used for the isolation of other yeast HAT complexes. In particular, the purification of the SAGA, ADA, SLIK, SALSA, NuA3 and NuA4 complexes start from Ni^2+^-NTA agarose fractionated whole cell extracts, as these enzymes fortuitously bind to this resin [9,10,13,14,17,22].

Most histone acetyltransferases have substrate specificities that direct the acetylation of specific residues within one or more of the core histones [5]. However, these substrate specificities are not fixed and can be altered by the association of the catalytic subunits with different protein complexes [19,49]. The presence of numerous HAT complexes expands the repertoire of modification states that can be generated on the chromatin template. Therefore, as growing evidence indicates that specific cellular processes are associated with precise patterns of histone modification, the presence of multiple HAT complexes in cells is likely to be a reflection of the myriad events that must take place in the context of chromatin [50].

Despite the importance of histone acetylation in regulating chromatin structure, with the exception of Esa1p, none of the yeast histone acetyltransferases are essential for viability [51,52]. Also, the deletion of most HAT genes results in only relatively mild phenotypes [35,36,53-57]. One explanation for this observation is that some HATs perform functionally redundant roles in the cell [58,59]. Alternatively, examination of the HAT activity profiles of fractionated extracts derived from HAT deletion strains presented here suggests that there may be mechanisms that can compensate for the lack of one histone acetyltransferase by increasing the activity of other HAT complexes. For example, in a Δ*sas2 *strain, there is a dramatic increase in an activity present in nuclear extracts that acetylates free histone H4 and which elutes from a DEAE column at a salt concentration similar to that of the nuclear form of Hat1p (Figure 3). In addition, deletion of the *HAT1 *gene causes a large increase in an activity that is coincident with the HatB3.1 activity. These results suggest the possibility that cells may monitor levels of histone modification and adjust specific HAT activities accordingly.

HatB3.1 is the third native HAT complex identified from yeast that is only capable of acetylating free histones [15,36]. In addition to the histone H4 specific Hat1p/Hat2p complex, the SAS complex, composed of Sas2p, Sas4p and Sas5p, was recently shown to acetylate free histones H3 and H4. The potential classification of the SAS complex as a type B HAT is supported by the fact that the SAS complex has also been shown to be physically associated with the histone deposition proteins Cac1p and Asf1p [16,60,61]. However, the specific target of SAS complex acetylation, histone H4 lysine 16, has not been found to be acetylated in the pool of newly synthesized histones in any organism [15,30]. Therefore, it remains to be determined whether the SAS complex participates in the acetylation of newly synthesized histones H3 and H4 prior to histone deposition or whether it is involved in the post-assembly modification of histones.

Gcn5p is the prototypical type A histone acetyltransferase. While rGcn5p is only capable of acetylating free histones under most experimental conditions, it has been identified as the catalytic subunit of five native HAT complexes that acetylate nucleosomal substrates (SAGA, ADA, A2, SLIK and SALSA) [9-11,13,14,31,62]. The most straightforward interpretation of the dependence of the HatB3.1 activity on a functional *GCN5 *gene and the co-elution of Gcn5p with highly purified HatB3.1 is that Gcn5p is also the catalytic subunit of HatB3.1. In the context of the type A HAT complexes, the Ada2p, Ada3p and TAF_II_68 proteins have been shown to be important for expanding the substrate specificity of Gcn5p to allow for the acetylation of nucleosomal histones [49,63-68]. Hence, the ability of Gcn5p to acetylate histones in chromatin is a property that must be conferred upon it by association with other proteins. The identification of Gcn5p as a component of a type B histone acetyltransferase activity suggests that classification as either type A or type B may not be an inherent property of an enzyme but, rather, may be a function of the association of the enzyme with specific accessory factors.

Several properties of HatB3.1 indicate that it is distinct from previously identified Gcn5p-containing complexes. First, HatB3.1 is the only native Gcn5p-containing complex that does not have detectable activity on nucleosomal substrates. Second, the apparent molecular weight of HatB3.1 (~500 kDa), as determined by size exclusion chromatography, is much lower than that of the SAGA, ADA, SALSA and SLIK complexes but is similar to that reported for the A2 complex [9,13,14,64]. However, unlike HatB3.1, the A2 complex is both dependent upon, and co-purifies with, Ada2p. These results clearly distinguish HatB3.1 as a novel Gcn5p-containing HAT complex [64].

Ada2p, Ada3p and Gcn5p form a module that provides the catalytic activity to their associated type A HAT complexes [5]. In these complexes, there does not appear to be any direct physical interaction between Ada3p and Gcn5p but, rather, their association is mediated through Ada2p [55,67,69,70]. The absence of Ada2p from the HatB3.1 activity suggests that Ada3p and Gcn5p can directly associate under certain circumstances or that another subunit(s) of the HatB3.1 complex can replace the function of Ada2p in bridging the interaction of Ada3p and Gcn5p. The identification of a Gcn5p-containing complex that is independent of Ada2p also suggests that there are cellular processes, such as histone deposition, that are influenced by Gcn5p (and Ada3p) but that do not require Ada2p. However, with the exception of the specific synthetic lethality seen with Δ*gcn5 *Δ*sas3 *mutants, deletions of the *GCN5*, *ADA2 *and *ADA3 *genes have similar *in vivo *consequences [22,59,71,72]. The absence of phenotypes unique to Δ*gcn5 *and Δ*ada3 *mutants may be the result of the complex functional redundancies observed in the assembly of chromatin. For example, Δ*hat1 *and Δ*hat2 *mutants only display phenotypes when combined with mutations in multiple lysine residues in the histone H3 NH_2_-terminal tail [45,46]. Uncovering these redundancies and deciphering the potential role of Gcn5p in the acetylation of newly synthesized histones is likely to require the characterization of the complete set of complexes that display type B histone acetyltransferase activity.

## Conclusions

In conclusion, we have fractionated yeast cytoplasmic and nuclear extracts and resolved several putative histone H3-specific type B histone acetyltransferase activities. One of these activities, HatB3.1, is highly specific for histone H3 that is free in solution. A combination of genetic and biochemical evidence indicates that HatB3.1 is a novel complex that depends on *GCN5 *and *ADA3 *but that is independent of *ADA2*.

## Methods

### Yeast strains

UCC1111 was used as the wild type yeast strain that serves as the genetic background for all deletion strains [45]. Null mutants for *GCN5*, *SAS3*, *SAS2*, *ADA2*, *ADA3 *and *HAT1 *were constructed using PCR-mediated gene disruption with the *HIS3 *reporter gene [73].

### Extract preparation

Cells were grown to mid-log phase in 1% yeast extract, 2% peptone, 2% glucose and 50 μg/mL ampicillin at 30°C. Cells were harvested at 4000 × g, 10', 4°C and total grams of cells recorded. All buffers contain 1.0 mM PMSF. Spheroplasts were prepared essentially as described previously using 0.25 mg of Zymolyase (U.S. Biologicals) per gram of cells for spheroplasting [74]. Spheroplasts were burst in 0.5 mL/g cells Lysis Buffer (18% Ficoll 400, 10 mM HEPES [pH 6.0]) followed by dilution in 1.0 mL/g cells Buffer A (50 mM NaCl, 1.0 mM MgCl_2_, 10 mM HEPES [pH 6.0]). Supernatant from a 1500 × g, 15' spin at 4°C was retained as a cytosolic extract. Pelleted material was washed once with Buffer A then resuspended in DN(1000) (DN buffers contain 25 mM Tris [pH 7.5], 10% glycerol, 0.1 mM EDTA and mM [NaCl] listed in parentheses). Supernatant from another 1500 × g spin as above yielded the nuclear extract. This extract was dialyzed O/N at 4°C into DN(0) to a conductivity similar to that of the cytosolic extract. Extracts were cleared by high speed centrifugation (~30,000 × g) prior to their chromatographic fractionation.

### Extract fractionation

All columns were equilibrated with and run using DN Buffers. HPLC (ÄKTA *purifier *– Pharmacia) was employed for all column runs.

#### Anion and cation exchange chromatography

DEAE – Cleared extracts were loaded onto a HiPrep 16/10 DEAE FF column (Pharmacia). Following a 5 C.V. wash with DN(50), proteins were eluted with a linear, 20 C.V., salt gradient from 50 mM to 1.0 M NaCl. A flow rate of 1.0 mL/min. was used and 3.0 mL fractions were collected.

CM – Either pooled peak fractions, dialyzed into DN(0) until at similar conductivity as DN(50) start buffer, or Flowthrough from the DEAE were loaded onto a HiPrep 16/10 CM FF column (Pharmacia). The column was washed and proteins eluted as described above.

Mono Q – The flowthrough fraction from the CM column was loaded onto a Mono Q HR 5/5 column (Pharmacia). Following a 5 C.V. wash with DN(50), a 20 C.V., linear, salt gradient was employed as above and 0.5 mL fractions were collected.

#### Ammonium sulfate precipitation

Peak fractions of HatB3.1 activity from the Mono Q column were pooled and brought to 75% (NH_4_)_2_SO_4 _(0.516 g/mL) over 30' at 4°C. Following an additional 30' equilibration period at 4°C, precipitated protein was pelleted (10,000 × g, 10', 4°C) and resuspended in 300 μL cold, DN(0).

#### Gel filtration chromatography

A 250 μL aliquot of resuspended ammonium sulfate precipitate was loaded onto a Superose 6 HR 10/30 column (Pharmacia). The column was equilibrated with and run in DN(350) at a flow rate of 0.3 mL/min. and 0.25 mL fractions were collected. Molecular weight standards (Sigma, MW-GF-1000) were run using the same parameters and 24 μL aliquots of every other fraction run on a 10% SDS-polyacrylamide gel. The elution profile of the MW standards was determined by protein visualization via Coomassie blue staining.

### Liquid HAT assays

Chicken erythrocyte core histones and chromatin were isolated as previously described [75,76]. Typically 10 μL aliquots of column fractions were incubated with 0.1 μM ^3^H-Acetyl Coenzyme A (5.50 Ci/mmol, Pharmacia) and ~1.0 mg/mL core histones or chromatin in a final volume of 100 μL at 1X [DN(75)]. 50 μL of each reaction was analyzed for HAT activity via liquid scintillation counting. The remaining assay mixture was brought to 1X [SDS Load Dye] to stop the reaction. In general, aliquots (24 μL) of these remaining assay mixtures were run on 18% SDS-polyacrylamide gels to resolve the histones. Gels were incubated in Autofluor (National Diagnostics), dried down and acetylated histone species visualized via fluorography.

### Western blot and gel analysis

Superose 6 fractions exhibiting HAT B3 activity, as determined above, were run on 10% SDS-polyacrylamide gels and proteins were either visualized by silver staining or transferred to nitrocellulose using a semi-dry transfer apparatus (Biorad). Blots were processed following standard procedures. Goat, polyclonal antibodies against Gcn5p, Ada2p and Ada3p (Santa Cruz Biotechnology, Inc.) were used at 1:100 dilutions in 5% Milk/TBS-T. Donkey, HRP-labeled Anti-Goat IgG secondary antibody (Santa Cruz Biotechnology, Inc.) was used at 1:2500 dilution followed by detection with ECL+Plus (Pharmacia) and visualization via phosphoimager (STORM 860, Pharmacia).

## Authors' contributions

A.R.S. performed all of the experiments presented here and drafted the manuscript. M.R.P. directed the project and edited the manuscript.
